# Nontyphoidal Salmonella as a Cause of Mediastinal Abscess in a Patient With Extensive Cardiac Surgery

**DOI:** 10.7759/cureus.9924

**Published:** 2020-08-21

**Authors:** Briana Janelle Dohogne, Sharanyah Srinivasan, Hina Arif-Tiwari, Anil Potharaju

**Affiliations:** 1 Medical Education, University of Arizona College of Medicine, Tucson, USA; 2 Internal Medicine, University of Arizona College of Medicine, Tucson, USA; 3 Radiology, University of Arizona College of Medicine, Tucson, USA

**Keywords:** salmonella, sepsis, mediastinitis, tetralogy of fallot

## Abstract

Focal infections caused by nontyphoidal Salmonella (NTS) are relatively rare and usually self-limited. Those with cardiac surgical history are predisposed to intrathoracic seeding, including mediastinal infections and abscesses. We report a case of a 39-year-old Hispanic male with a complex past medical history of Tetralogy of Fallot with an initial presentation of Salmonella gastroenteritis and concern for sepsis. The patient did not clinically improve on ceftriaxone despite appropriate cultures and susceptibilities, and another source of infection was speculated. A chest CT scan showed development of a mediastinal abscess with compression of the right ventricular outflow tract. The patient was not deemed an appropriate surgical candidate and was managed conservatively on ceftriaxone and ciprofloxacin. He was discharged in stable condition. This case of NTS infection associated with a mediastinal abscess is a rare occurrence, and management is complicated. To improve morbidity and mortality, early imaging is essential to diagnose distal seeding of the infection in patients with enteral infections who do not show clinical improvement despite appropriate antibiotic treatment. Surgery is the standard of care, but conservative management might be required in certain high-risk cases.

## Introduction

Nontyphoidal Salmonella (NTS) are gram-negative bacilli that predominantly cause intra-abdominal infections, the most common being gastroenteritis. Appendicitis and cholecystitis have also been observed [1]. Focal infections are usually self-limited. However, in the elderly and the immunosuppressed, bacteremia can develop [2]. Mediastinal infections with NTS are atypical, with only a handful of cases described. Case reports have detailed pre-existing risk factors, such as antecedent tissue disruption, immunocompromised state, atherosclerosis, and food or animal exposure. Postoperative mediastinal infections in the context of congenital heart disease are rarer still, with reports mainly occurring in the pediatric age group [3]. We present a case of NTS-induced mediastinal abscess in an adult male, status post a series of congenital cardiac repairs in the past.

## Case presentation

A 39-year-old Hispanic male from the department of corrections (DOC) with a complex cardiac history presented to the hospital for one week of watery diarrhea associated with fevers, sweats, nausea, vomiting, dyspnea, and chest pain. No other inmates had similar symptoms, and the patient reported eating prepared meals from an outside delivery service.

The patient’s medical history was significant for Tetralogy of Fallot status post right ventricular pulmonary arterial (RV-PA) conduit in childhood complicated by multiple conduit replacements, history of prosthetic pulmonic valve endocarditis, atrial fibrillation, atrial flutter treated with multiple catheter ablations, heart failure with reduced ejection fraction, hypertension, and dual-chamber pacemaker placement. He denied a history of Clostridium difficile infection, antibiotic or antacid use, sick contacts, animal contact, or anal penetration. He quit smoking two years ago after a 15-year history, and his tuberculosis testing six months prior was negative. Tuberculosis testing was completed in accordance with annual testing guidelines within the DOC. 

On presentation, the patient was febrile, tachypneic, and hypotensive but responded to fluid resuscitation. Electrocardiogram on admission showed atrial paced complexes, but no new abnormalities. Laboratory values were remarkable for leukocytosis, but comprehensive metabolic panel, urinalysis, and lactate were unremarkable. A chest X-ray showed cardiomegaly without consolidation, effusion, or pneumothorax. Blood cultures were obtained, and the patient was started on empiric parenteral antibiotics for suspected bacterial gastroenteritis.

Blood cultures grew NTS species, and he was switched to intravenous ceftriaxone per susceptibilities. However, the patient continued to be febrile after three days of appropriate antibiotics and had worsening dyspnea. Due to his lack of clinical improvement, further imaging was obtained including an echocardiogram, given the patient’s extensive cardiac history, and a CT of the chest, abdomen, and pelvis due to the concern for distant seeding of the infection.

**Figure 1:**
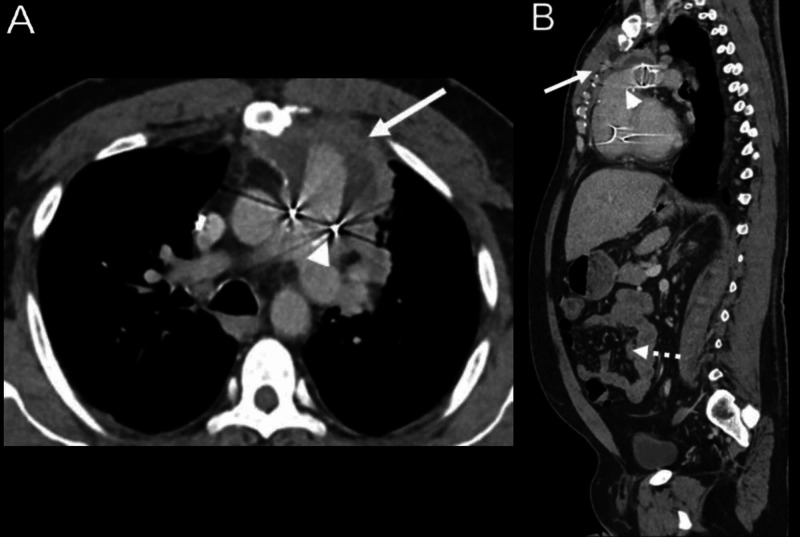
Contrast-enhanced CT of the chest, abdomen, and pelvis showing mediastinal abscess. Axial and sagittal postcontrast CT through the chest reveals pulmonary outflow tract reconstruction evident postsurgical material and resultant streak artifact (arrowhead). Anterior to the pulmonary artery outflow tract, hypodense rim enhancing fluid collection is seen suggestive of abscess (A and B, arrows). Mild thickening of mid small bowel loops with surrounding fat stranding is suggestive of enteritis (B, dashed arrow). Urinary bladder wall thickening and inflammatory changes represent cystitis.

The CT showed a complex loculated ring-enhancing fluid collection within the anterior mediastinum surrounding the reconstructed pulmonary artery outflow tract and abutting the posterior mediastinum with reactive mediastinal lymph nodes (Figure 1). A trans-thoracic echocardiogram confirmed the presence of a mobile, organized mass seen adjacent to the proximal RV-PA conduit (Videos 1, 2). Although unchanged from previous studies, this mass was suspected to be the likely nidus for infection. The consensus reached by Pediatric Congenital and Adult Cardiology was to defer interventional radiology-guided drainage and surgical intervention in favor of medical management due to anterior mediastinal location of the fluid, the patient’s extensive cardiac history, including five sternotomies, and risk of complications. Intravenous ceftriaxone was continued for a planned duration of six weeks.

**Video 1:** Parasternal right ventricular outflow tract (RVOT) sweep of anterior mediastinal mass.

**Video 2:** Parasternal right ventricular outflow tract (RVOT) Doppler.

After being afebrile for 48 hours on the antibiotic regimen, the patient’s clinical course was complicated by recurrent fevers, chills, and new-onset hemoptysis. A repeat CT scan of the chest now revealed a focus of gas around the previously identified fluid collection, and metronidazole was added for anaerobic coverage. Repeat blood cultures done at this time continued to be negative, an echocardiogram did not reveal any vegetations concerning for endocarditis, and a laryngoscopy did not identify a bleeding source. Bronchoscopy could not be attempted due to the patient’s increased anesthetic risk. Due to persistent fevers and hypotensive episodes, the antibiotics were broadened. 

**Figure 2:**
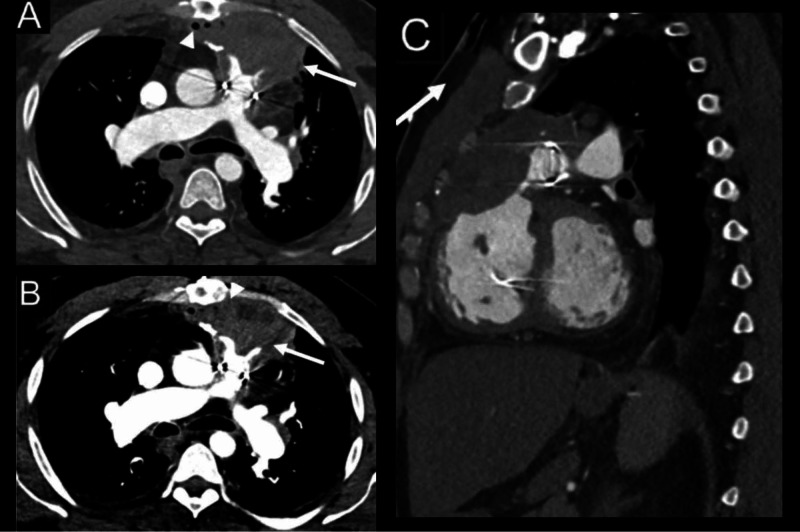
CT pulmonary angiogram of the chest showing the mediastinal abscess with a superimposed hematoma. Axial CT pulmonary angiogram shows a marked increase in size of the anterior mediastinal lesion (A, arrow), which causes encroachment and mass effect on the pulmonary artery outflow tract without active contrast extravasation. Air foci in the anterior mediastinum represent ongoing infection (A, arrowhead). On narrow window settings, hyperdense hematoma is seen abutting the pulmonary outflow tract (B, arrow), in contrast to less dense anterior abscess (B, arrowhead). Expanding hematoma extends to compress the right ventricular outflow tract (C, arrow).

The patient’s condition further deteriorated due to massive hemoptysis, hypoxia, and hemodynamic instability requiring monitoring. A CT angiogram of the chest showed a worsening of the anterior mediastinal abscess with a superimposed hematoma that was exerting a significant mass effect on the reconstructed right ventricular outflow tract without extravasation (Figure 2). Although the right ventricular outflow tract obstruction was a serious concern, it was rendered moot by the patient’s likely inability to withstand cardiopulmonary bypass, surgical intervention, or extracorporeal membrane oxygenation.

The patient remained in intensive care for another week, was eventually weaned off oxygen, and the patient was discharged in a hemodynamically stable condition on ciprofloxacin and ceftriaxone for presumed Salmonella endocarditis. Serial echocardiograms on an outpatient basis were recommended after completion of the six-week oral antibiotic course. On follow-up in the clinic, the patient had an improvement in his symptoms without further chest pain, hemoptysis, fevers, or diarrhea. Serial echocardiograms showed no evidence of endocarditis. 

## Discussion

Mediastinal infections in patients who have undergone cardiac surgery are extremely rare with an incidence of 0.4% to 5.0% [4-6]. Although rare, they have a high mortality of 12.8% during hospital stay, increasing to 20.5% within one year [7]. In adults, the majority of studies regarding poststernotomy mediastinitis were in those undergoing coronary artery bypass grafts (CABG), valvular replacements, or a combination of the two [8]. Most cases of mediastinal infection following congenital heart surgery are described in the pediatric age groups. Although risk of postoperative infection is comparable in both populations, incidence in adults with remote congenital history is not well studied. 

NTS has been shown to have a predilection for the endovascular environment. Staphylococcus aureus and NTS are two of the most common pathogens isolated from mycotic aortic aneurysms [9]. NTS has also been documented to cause other cardiac complications, such as myocarditis, pericarditis, and endocarditis [10]. In our patient’s case, it is likely that he developed a gastrointestinal infection with NTS with subsequent distant seeding. There was initially a suspicion for endocardial involvement, but echocardiography was unremarkable for any vegetations before a CT scan of the chest confirmed that an abscess had formed, surrounding the reconstructed pulmonary artery. Mediastinal abscesses have been reported in literature with no other obvious source [11,12].

Imaging is critical to the diagnosis of infection and visualization of the mediastinum. These abscesses are usually described as low-attenuation masses with peripheral ring enhancement on CT imaging [3,13]. While echocardiogram might be the standard screening modality for endocarditis as a potential source of infection, this case serves to highlight that cross-sectional imaging should be considered if a history of previous cardiac surgery is present. CT can delineate abscesses more clearly and better than other intrathoracic structures. Additionally, it can also aid with biopsy, percutaneous intervention, and in planning a surgical approach [14]. 

The standard of treatment of mediastinal infections regardless of etiology includes aggressive surgical treatment supported by antibiotic therapy [11]. Surgical intervention is considered based on the extension and location of the abscess within the mediastinum and the perioperative risk. Surgery has been preferred in reports of Salmonella-induced mediastinal infection without local invasion as it is both diagnostic and therapeutic [12]. Localized suppuration, sepsis, and spread of infection to prosthetic materials used in cardiothoracic surgery are all grave complications [15]. Awareness of these complications may allow for their appropriate diagnosis and eventual prevention.

In patients with extensive prior cardiac surgical history, or inoperable location of infection as in our case, there is some evidence for successful treatment with antibiotics for these types of infections. The most used antibiotic reported is ciprofloxacin [16]. However, there is concern for resistance patterns in NTS. According to the CDC National Antimicrobial Resistance Monitoring System (NARMS) for enteric bacteria, NTS infections not susceptible to ciprofloxacin reached 74% in 2017 [17]. Fortunately, our patient’s antimicrobial susceptibility results showed responsiveness to ciprofloxacin. However, the increased resistance of NTS severely limits treatment options for future infections not amenable to surgical intervention. The two antibiotic classes currently recommended by the CDC for the treatment of drug-resistant Salmonella are macrolides and carbapenems. Relapsing bacteremia following a therapeutic antibiotic course should prompt further investigation to rule out drug resistance or other endovascular infections. In the latter, surgical intervention is mandated [18].

## Conclusions

Mediastinal infections with NTS are rare. In those with a history of cardiac surgery, cardiac source of infection is especially easy to assume, but early imaging with CT is essential to diagnose distal seeding in those who do not show clinical improvement despite appropriate antibiotic treatment. Surgery is the standard of care, but conservative management with long-term antibiotics might be required in certain high-risk cases.
